# Design Optimization of a Hybrid-Driven Soft Surgical Robot with Biomimetic Constraints

**DOI:** 10.3390/biomimetics9010059

**Published:** 2024-01-21

**Authors:** Majid Roshanfar, Javad Dargahi, Amir Hooshiar

**Affiliations:** 1 Surgical Robotics Laboratory (SRL), Department of Mechanical Engineering, Gina Cody School of Engineering, Concordia University, Montreal, QC H3G 1M8, Canada; m_roshan@encs.concordia.ca (M.R.); dargahi@encs.concordia.ca (J.D.); 2 Surgical Performance Enhancement and Robotics (SuPER) Centre, Department of Surgery, McGill University, Montreal, QC H3G 1A4, Canada

**Keywords:** soft robot, design optimization, hybrid-driven, minimally invasive intervention, finite element simulation

## Abstract

The current study investigated the geometry optimization of a hybrid-driven (based on the combination of air pressure and tendon tension) soft robot for use in robot-assisted intra-bronchial intervention. Soft robots, made from compliant materials, have gained popularity for use in surgical interventions due to their dexterity and safety. The current study aimed to design a catheter-like soft robot with an improved performance by minimizing radial expansion during inflation and increasing the force exerted on targeted tissues through geometry optimization. To do so, a finite element analysis (FEA) was employed to optimize the soft robot’s geometry, considering a multi-objective goal function that incorporated factors such as chamber pressures, tendon tensions, and the cross-sectional area. To accomplish this, a cylindrical soft robot with three air chambers, three tendons, and a central working channel was considered. Then, the dimensions of the soft robot, including the length of the air chambers, the diameter of the air chambers, and the offsets of the air chambers and tendon routes, were optimized to minimize the goal function in an in-plane bending scenario. To accurately simulate the behavior of the soft robot, Ecoflex 00-50 samples were tested based on ISO 7743, and a hyperplastic model was fitted on the compression test data. The FEA simulations were performed using the response surface optimization (RSO) module in ANSYS software, which iteratively explored the design space based on defined objectives and constraints. Using RSO, 45 points of experiments were generated based on the geometrical and loading constraints. During the simulations, tendon force was applied to the tip of the soft robot, while simultaneously, air pressure was applied inside the chamber. Following the optimization of the geometry, a prototype of the soft robot with the optimized values was fabricated and tested in a phantom model, mimicking simulated surgical conditions. The decreased actuation effort and radial expansion of the soft robot resulting from the optimization process have the potential to increase the performance of the manipulator. This advancement led to improved control over the soft robot while additionally minimizing unnecessary cross-sectional expansion. The study demonstrates the effectiveness of the optimization methodology for refining the soft robot’s design and highlights its potential for enhancing surgical interventions.

## 1. Introduction

Applications of soft robots in the field of minimally invasive surgery (MIS) have experienced considerable growth owing to their distinct capabilities, which arise from their compliant materials [1,2] typically exhibiting a Young’s modulus within the megapascal range [3]. The mechanical properties of the soft robots were found to exhibit a close resemblance to human skin [4,5], thereby introducing a heightened level of safety for surgical procedures. Considerable attention has been given to this domain due to the extensive array of soft materials that have become accessible in recent times [6], enabling the creation of highly agile robots equipped with capabilities surpassing those of their rigid counterparts [7]. In addition, soft robots have demonstrated promise across a wide range of applications [8,9], such as robot-assisted minimally invasive surgery (RAMIS) [10,11], cardiac mapping catheters [12], soft sensors [13,14,15,16], cardiac ablation [17], endovascular treatment [18], and rehabilitation [19,20], because they are inherently safe [21] and are better at absorbing dynamical loads and shock [22]. The medical field recognized the need for the creation of instruments that could operate with efficiency in environments characterized by a lack of structure and constant change while also being able to navigate obstacles [23]. It became highly desirable for a robotic structure to have the ability to regulate its stiffness, i.e., the ability to transition easily from a soft state, allowing for easy insertion, to a stiff state, facilitating the transmission of force upon reaching the intended target tissue. As a response to this demand, hybrid-driven robotic systems have emerged as a promising and effective solution [24], enhancing the performance of each individual actuation. One drawback of hybrid-driven systems is that their increased complexity may pose challenges in terms of system control and potential risks associated with the complex integration of different actuation technologies. Balancing these advantages and limitations is essential for optimizing the overall performance and usability of hybrid-driven robotic systems.

Extensive research has been conducted in the field of soft robotics to explore various types of actuation mechanisms. Among these mechanisms, fluid actuation, specifically pneumatic [25] and hydraulic [26] actuation, has received considerable attention due to its various advantages, including its lightweight construction and high output torque. However, the drawbacks linked to fluid-driven mechanisms include the complexity of the control system and the potential risk of leakage into the blood vessels [27]. These systems are characterized by a substantial volume and weight, creating significant challenges in the effort to miniaturize the entire soft manipulator system [8]. The use of cable-driven mechanisms [28,29,30] has been investigated as an alternative actuation mode. It allows for the transmission of driving forces over long distances which, in turn, ensures a low moment of inertia for the manipulator. However, the implementation of cable-driven actuation presents challenges in achieving miniaturization, primarily due to the necessity of motors and cable retracting and releasing devices. On the other hand, electroactive polymer (EAP)-driven actuation has gained attention due to its various characteristics, including its lightweight construction [31], high energy density, and ease of miniaturization. Nonetheless, this mode also faces specific challenges, such as the risk of material damage from high-level voltage, the limited load capacity, and the low control accuracy. Another potential actuation mode is based on shape memory materials, which offer a miniaturized structure with a relatively sufficient torque [32]. However, this mode relies on phase changes caused by heating, resulting in a slower response. These limitations hinder its control accuracy and restrict its potential application areas. Within the medical field, electromagnetic-driven actuation has demonstrated promise. Recently, the millimeter-scale miniaturization of electromagnetic-driven soft robots has been achieved [33]. While the potential of magnetic actuation appears promising [34,35], this mode of actuation necessitates the use of complex external equipment to generate a stable magnetic field for motion control.

To overcome the limitations associated with individual actuation modes, researchers have conducted investigations into a hybrid-driven mode. This mode involves the combination of two or more different drive modes to enhance the load capacity and control accuracy of the soft manipulators. One example of such a hybrid-driven mode is the combination of the fluid-driven mode and the cable-driven mode. The fluid-driven mode demonstrated a favorable characteristic in terms of pressure bearing but exhibited a relatively weak performance for tensile bearing. In contrast, the cable-driven mode offered robust tensile resistance but lacked the ability to withstand pressure. By integrating these two distinct drive modes, soft manipulators can benefit from both pressure-bearing and tensile-bearing properties, resulting in an increased load capacity and an improved position control accuracy. Overall, hybrid modes combine the complementary strengths of different actuation mechanisms. For instance, Kang et al. [36] conducted a study on a pneumatic soft robot with embedded tendons, illustrating the application of hybrid actuation. Also, the research aimed to achieve a variable stiffness through hybrid actuation, drawing inspiration from the approaches explored by Yin et al. [37] in their design of a soft gripper with a wide range of tunable stiffnesses. They achieved this objective by employing a hybrid actuation system that integrated tendon-spring and air pressure mechanisms. Similarly, Shahid et al. [38] developed a soft composite finger with an adjustable joint stiffness using a hybrid actuation approach combining cable tension and air pressure. Furthermore, Roshanfar et al. [39,40,41,42] developed a continuum model of a tendon–air hybrid actuated soft robot based on the Cosserat rod model that was specifically designed for applications in the RAMIS.

**Figure 1:**
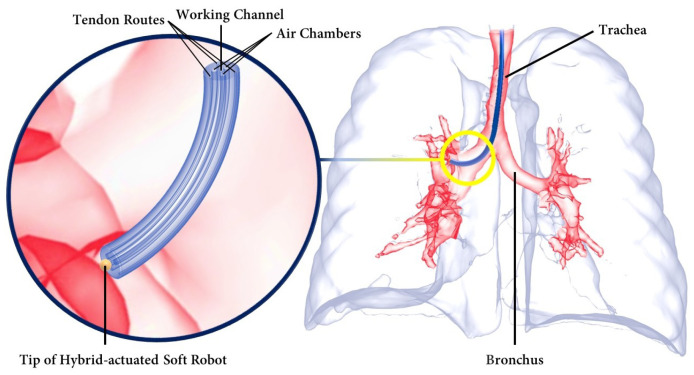
Hybrid-driven soft surgical robot inside the lungs during an intra-bronchial intervention.

Inspired by nature [43], soft robots have provided innovative solutions for medical interventions [44]. Within the medical field, soft robots have found wide applications as flexible tubes, commonly referred to as catheters, for MIS. While the bodies of the catheters are made of soft and flexible materials such as Pebax, the presence of rigid metallic materials in the tips of the catheters, serving as end effectors, has the potential to harm patients during manipulation [45]. This is attributed to the higher Young’s modulus of the metallic tips compared to that of human vessels, which can apply an unnecessary excessive contact force [10]. As an alternative, soft robots have been proposed to replace or enhance the existing catheters in MISs, such as cardiothoracic endoscopic surgery [46], abdominal surgery [47], and bronchoscopy [48]. Figure 1 illustrates a hybrid-driven soft surgical robot used for intra-bronchial interventions, which serves as a representative use case.

**Figure 2:**
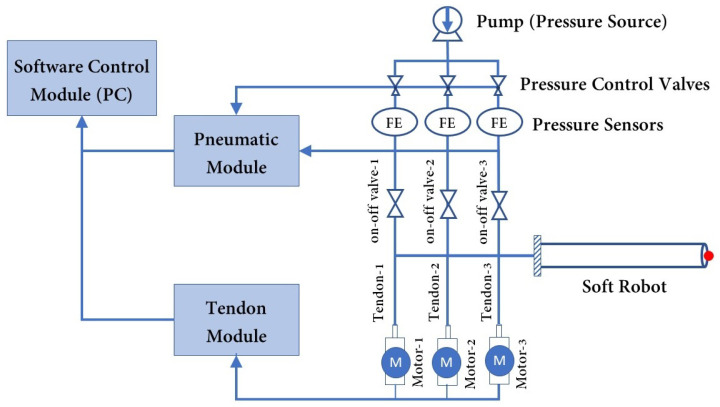
Setup architecture of the hybrid air–tendon-driven soft robot for use in RAMIS.

In terms of definition, a soft robot can be described as a continuum robot that is capable of bending continuously, providing virtually infinite degrees of freedom (DOF) [49]. Several studies have been carried out to investigate the kinematics and dynamics of soft robots [50,51]. Subsequently, control analyses were performed using mechanistic-based models as well as learning-based models [52]. However, designing an optimal soft robot remains a challenging task in the field. One significant challenge lies in achieving the ideal combination of structural strength and flexibility. Soft robots need to maintain a robust form while still being flexible enough to navigate complex environments or perform complex tasks. Achieving this balance necessitates thorough experimentation with different design approaches to overcome the inherent trade-offs between rigidity and maneuverability. The current study focused on optimizing the geometry of catheter-like pneumatically actuated tendon-driven soft robots for use in RAMIS. To achieve this, a cylindrical soft robot with three air chambers, three tendons, and a central working channel was considered. The cylindrical shape has been proven to be the most effective geometry for intra-vessel insertion applications. The dimensions of the soft robot, including the air chambers’ lengths, the diameters of the air chambers, and the offsets of the air chambers and tendons, were optimized to minimize the goal function. Developing an optimized model for soft robots is an iterative process that begins with the definition of objectives, variables, and constraints [53]. Section 2 provides the necessary requirements for the design optimization of a hybrid-driven soft robot, taking into account the constraints associated with each specific surgical task. Additionally, the material modeling of silicon for finite element analysis (FEA) simulations is discussed. Following the presentation of results and discussion in Section 3, an example of a robot-assisted intervention using the optimized geometry of the hybrid-driven soft robot is provided as a representative case. Moreover, to ensure the clarity of the hybrid-driven system, the architectural design of the hybrid air–tendon-driven soft robot is depicted in Figure 2. This design consists of two distinct modules, the pneumatic module and the tendon module, both of which are interconnected with the software control module. Each tendon was independently actuated by its own DC brushless motor, and each air chamber was activated separately and controlled using a pressure controller. The tension in the tendons was measured by evaluating the torque exerted on the pulley attached to the motor shaft, while the pressure levels were directly monitored through pressure sensors.

## 2. Materials and Methods

### 2.1. Optimization Model

#### 2.1.1. Design Objectives

The design objective of this study was to achieve the desired mechanical behavior of a hybrid-driven soft robot. Specifically, the investigation focused on one-degree bending deformation of the soft robot, taking into account the effects of a single air chamber actuation and the corresponding tendon tension. The primary aim was to minimize the required actuation effort, taking into account the input pressure and tendon tension while simultaneously reducing the radial expansion of the soft robot. By achieving 90° bending, the objective was to facilitate greater control over the robot’s deformation while reducing the cross-sectional expansion.

To accomplish this objective, a specific setup was employed. The soft robot’s deformation was controlled by inflating a single air chamber while simultaneously pulling a single tendon, thus causing bending in a two-dimensional (2D) plane. It is important to note that this particular objective can be described as a form of “multi-objective optimization”, given the simultaneous consideration of multiple variables. In order to mathematically formulate this objective, various parameters were taken into account. These included the input air pressure, tendon tension, and the radial expansion of the soft robot. By formulating the objective in this manner, the study aimed to establish an optimal configuration to achieve the desired mechanical behavior while minimizing the goal function. The goal function was formulated as follows:

(1) $$G=w\left[{\left(\frac{P_{ch}}{P_{max}}\right)}^{2}+{\left(\frac{T_{t}}{T_{max}}\right)}^{2}\right]+\left(1-w\right){\left(\frac{A}{A_{0}}-1\right)}^{2}$$

where *G* represents the goal function to be minimized by the optimal geometry. Additionally, the variable *w* is used to denote the weight factor ( 0⩽w⩽1 ), which determines the relative significance of the actuation effort (the first term in (1)) or radial expansion (the second term in (1)) based on the specific surgical application. The air pressure within the chamber of the soft robot is denoted by $P_{ch}$ , while the tension in each tendon is represented by $T_{t}$ , which is connected to the tip of the soft robot. The maximum values that can be obtained from the hardware, namely the air pump and motors, are defined as $P_{max}$ and $T_{max}$ , respectively. Also, $A_{0}$ and *A* are the cross-sectional areas of the soft robot before and after the deformation, respectively. The inclusion of the ${\left(A/A_{0}-1\right)}^{2}$ term in (1) was necessary for two reasons. Firstly, a previous study [41] demonstrated that the Cosserat rod model employed to describe the deformation of the soft robot did not account for the radial expansion of the soft robot during inflation. As the pressure inside the air chambers increased, the disparity between the model and experimental results also increased. Therefore, the ${\left(A/A_{0}-1\right)}^{2}$ term was introduced to capture this radial expansion effect. Secondly, the absence of this term would have made the solution to the optimization problem obvious. Without considering the impact of the radial expansion, increasing the offset of air chambers and tendons near the wall edge would have led to a minimal actuation effort. By including the ${\left(A/A_{0}-1\right)}^{2}$ term, the optimization problem becomes meaningful and realistic, accounting for the trade-off between the actuation effort and radial expansion during the soft robot’s deformation.

#### 2.1.2. Design Variables

**Figure 3:**
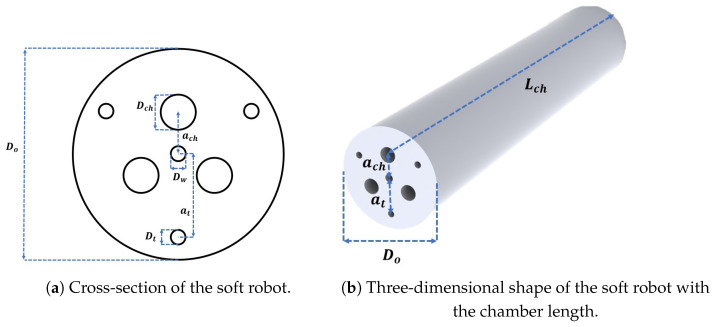
Design variables (**a**) $D_{w}$ represents the diameter of the working channel, $D_{ch}$ is the diameter of the air chambers, $D_{t}$ is the diameter of the tendon passes, $D_{o}$ is the outer diameter of the soft robot, and $a_{ch}$ and $a_{t}$ represent the offsets of the air chambers and tendon passes from the center of the cross-section, respectively. (**b**) $L_{ch}$ represents the length of the air chambers.

Generally, design variables refer to the geometry, material, and type of actuation employed in the development of a hybrid-driven soft robot [53]. In this study, the material and type of actuation remained constant throughout the optimization process. Consequently, it was essential to optimize the soft robot’s geometry, including parameters such as the length and diameter of the air chambers and the offests of the chamber and tendon passes from the center to satisfy the desired design objective. To comprehensively explore the design space including all variables, it was necessary to consider all feasible design candidates for each particular task. For the sake of clarity, Figure 3 illustrates the cross-sectional variables and chamber length of the hybrid-driven soft robot for this study. Within the soft robot’s cross-section, $D_{w}$ denotes the diameter of the working channel, $D_{ch}$ represents the diameter of the air chambers, $D_{t}$ corresponds to the diameter of the tendon passes, and $D_{o}$ is the outer diameter of the soft robot. The outer diameter of the soft robot was assigned a specific value, dictated by the requirements of each clinical intervention. Due to this, its value remained the same throughout the iterations to find the optimal geometry. Furthermore, $a_{c}h$ and $a_{t}$ denote the offsets of the air chambers and tendon passes, respectively, from the center of the cross-section. Additionally, $L_{o}$ and $L_{ch}$ represent the outside length of the soft robot and the length of the air chambers, respectively. Throughout the optimization process, the lengths of all three air chambers remained equal, implying that $L_{ch}^{\left(1\right)}=L_{ch}^{\left(2\right)}=L_{ch}^{\left(3\right)}$ . Additionally, the diameters of all three air chambers and the diameters of all three tendon passes remained equal, namely, $D_{ch}^{\left(1\right)}=D_{ch}^{\left(2\right)}=D_{ch}^{\left(3\right)}$ and $D_{t}^{\left(1\right)}=D_{t}^{\left(2\right)}=D_{t}^{\left(3\right)}$ . Moreover, the length of the tendon passes was equal to $L_{o}$ , ensuring that the tendons were consistently affixed to the tip of the soft robot. The number of air chambers and tendons was also fixed at three, and these were evenly distributed within the cross-section, as a minimum of three inputs is required to maneuver the soft robot’s tip within a 3D space.

#### 2.1.3. Design Constraints

To ensure the effective utilization of soft robots in interventional medical applications, the consideration and resolution of two distinct categories of requirements were found to be essential: technical requirements and clinical requirements. Furthermore, it was observed that soft robots employed in interventional medical applications must conform to the unique demands and specifications associated with diverse clinical procedures. This involves the careful examination of factors such as the dimensions and shapes of anatomical structures, the nature of the interventions being conducted, and the expected outcomes. Accommodating various clinical scenarios necessitates the incorporation of modularity and customization options, thereby enabling the customization of soft robots to suit specific medical interventions and patient requisites.

##### Technical Requirements

Technical requirements cover a broad range of factors related to the design, functionality, and operation of soft robots. These soft robots must possess specific mechanical properties that enable them to perform precise movements within the human body. Key attributes such as flexibility, compliance, and adaptability are essential for soft robots to navigate complex anatomical structures without causing damage or discomfort. In particular, the technical requirements include the engineering constraints and manufacturing limitations. For instance, it has been observed that when using a normal 3D-printed mold approach to prototype the soft robot, the minimum diameter of a cavity should be at least 1 mm [39]. Therefore, the following condition holds:

(2) $$\begin{array}{c} D_{ch}⩾1\;\mathrm{mm} \end{array}$$

(3) $$\begin{array}{c} D_{t}⩾1\;\mathrm{mm} \end{array}$$

(4) $$\begin{array}{c} D_{w}⩾1\;\mathrm{mm} \end{array}$$

Moreover, for effective inflation and to prevent the “ballooning effect”, alternative research proposes the restriction of the outer diameter of the soft robot using rigid constraints [54] or fibers [55]. Nevertheless, evidence indicates that these external constraints can limit and change the deformation mode of the soft robot. In the present study, the geometry of the soft robot was optimized to minimize unnecessary radial expansion and avoid the ballooning effect. From a manufacturing perspective, soft robots should have a minimum wall thickness of 1 mm [56,57]. This can be expressed as follows:

(5) $$t_{w}=R_{o}-R_{t}-a_{t}⩾1\;\mathrm{mm}$$

where $t_{w}$ represents the wall thickness, and $R_{o}$ and $R_{t}$ denote the outer and tendon pass radii, respectively. Similarly, there should be at least a 1 mm space between the working channel and the air pressure and tendon passes. By referring to these technical requirements, the design and fabrication of soft robots can be optimized, ensuring their feasibility for prototyping.

##### Clinical Requirements

Clinical requirements refer to the operational capabilities of a soft robot within a specific surgical procedure. Surgical instruments are typically subject to constraints in their physical dimensions due to the nature of their operating environment. For example, MISs involving the oral cavity and esophagus necessitate a surgical robot with a feature size of less than 30 mm in diameter [58]. Similarly, intra-vascular procedures require a diameter of less than 6 mm [10], while endoscopic applications mandate an instrument diameter of less than 15 mm [59]. Enhanced miniaturization, which is closely linked to the soft robot’s capacity for greater penetration into vessels, enables the robot to navigate more deeply and extensively within confined spaces. Throughout this study, the outer diameter of the soft robot was assumed to be constant:

(6) $$D_{o}=15\;\mathrm{mm}$$

while it is essential for a soft robot to be of adequate length for intra-luminal procedures, e.g., typically ranging from 1.5 to 2 m [6], only the active tip of the robot was studied:

(7) $$L_{o}=84\;\mathrm{mm}$$

which was 80 mm for the body and 4 mm for the cap. To adapt to the majority of the commercially available endoscopic micro-cameras, (e.g., OdySight.AI, Ramat Gan, Israel), the internal diameter of the working channel was also fixed at 1.2 mm. For different applications, the endoscope’s field of view (FOV) may vary. Currently, for upper and lower gastrointestinal endoscopies, the standard FOV is 170°. However, in applications where the endoscope is constrained to a narrow lumen with no possibility of moving away from the longitudinal axis, cameras with a FOV of 120° or less are commonly used and provide adequate vision [3]. In the current study, the minimum required bending angle θ of the soft robot, based on clinical requirements, was selected to be 90°, combined with a 120° FOV, which would adequately cover a total FOV of 360°.

(8) $$\theta ⩾90^{\circ }$$

### 2.2. Material Modeling

Silicone rubber is the most commonly used material for soft pneumatic actuators, as it is highly flexible and can handle large strain values. To adequately characterize their mechanical behaviors, hyperelastic models are employed. In this study, silicone rubber was considered to be isotropic and incompressible, while inelastic phenomena such as viscoelasticity and stress-softening were neglected [60]. The third-order Yeho model was selected, because it is applicable to a much wider range of deformation and can predict the stress–strain behavior in different modes based on data collected from one simple uniaxial test. According to the Yeoh model, the strain energy density function is [61]:

(9) $$W=\sum_{i=1}^{3}C_{i0}{\left(I_{1}-3\right)}^{i}$$

where the energy function *W* is defined as the amount of elastic energy stored in a unit volume of material, $C_{i0}$ are the material constants, and $I_{1}$ is the principal invariant:

(10) $$I_{1}=\lambda^{2}+\frac{2}{\lambda }$$

where λ indicates the principal stretches that represent the deformation of a differential cubic volume element in relation to the principal axes of a Cartesian coordinate system [62]. The corresponding stress–stretch function (principal Cauchy stresses) of (9) is:

(11) $$\sigma =2\left(\lambda -\frac{1}{\lambda^{2}}\right)\frac{\partial W}{\partial I_{1}}$$

**Figure 4:**
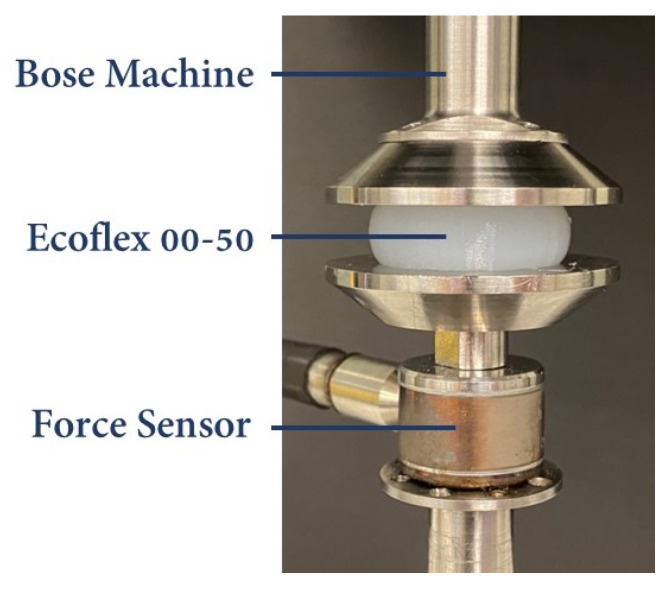
Compression test performed with Bose UTM on the Ecoflex-50 samples based on ISO 7743.

**Figure 5:**
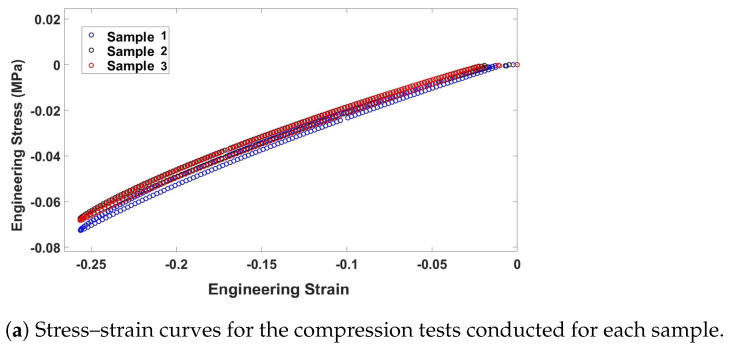

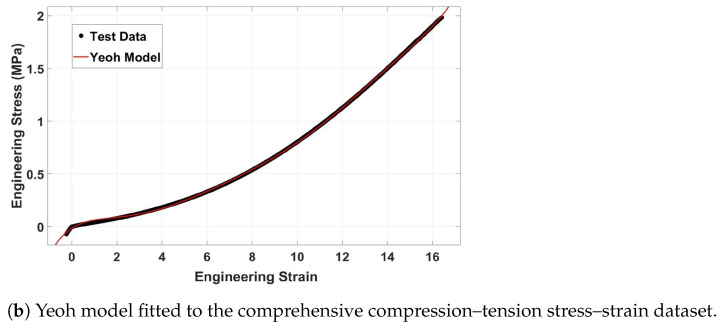
(**a**) Engineering stress–strain compression curve (**b**) comprehensive compression–tension engineering stress–strain curve for Ecoflex 00-50. The tension data extracted from [61].

The determination of the material properties, specifically $C_{10}$ , $C_{20}$ , and $C_{30}$ , as indicated in (9), requires the employment of uniaxial testing. In this study, the soft robot was subjected to simulation and prototyping by employing Ecoflex 00-50 (Smooth-On Inc., Macungie, PA, USA). To obtain the material constants, a uniaxial compression test was conducted on three samples, according to the guidelines outlined in ISO 7743 [63]. After the curing of the samples, their final dimensions fell within the tolerances specified in ISO 7743, with a diameter of 29 mm ± 0.5 mm and a height of 12.5 mm ± 0.5 mm. The compression test, depicted in Figure 4, was carried out using the Bose electroforce universal testing machine (UTM), following the protocols defined in ISO 7743. Each sample was subjected to a cyclic load, and the fourth cycle was utilized to extract the stress–strain curve. Figure 5a presents the stress–strain curves obtained from each sample utilized in this study. To establish a comprehensive compression–tension model for Ecoflex 00-50, the compression test results were combined with tension test data from L. Marechal et al. [61]. Figure 5b demonstrates the comprehensive compression–tension engineering stress–strain curve for Ecoflex 00-50. Finally, the material constants required for the Yeoh model in (9) were fitted using the combined dataset, and the resulting constants are presented in Table 1.

### 2.3. Design Optimization

There are several analytical approaches for modeling the deformation of soft robots, like the piecewise constant curvature (PCC) [50] or the Cosserat rod model [51]. However, due to the nonlinearity of the model, a finite element (FE) model was used to determine the deformation of soft robots under both actuation modalities. Then, the goal function at various design points was measured to find the optimum geometry based on the design constraints. To do so, the FE model of the soft robot with the initial dimensions was imported into the ANSYS^®^ software (R17.0, PA, USA). The dimensions described in Section 2.1.2 are considered to be parameters, so the geometry of the soft robot was updated automatically during each iteration. Next, the upper and lower limits for each individual input parameter were defined, and numerical parametric optimization was performed using the response surface optimization (RSO) module in ANSYS^®^ software, similar to what was conducted in a previous study [64].

**Figure 6:**
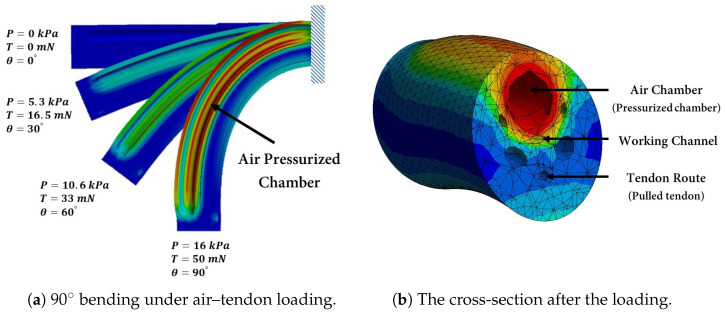
Deformation of the soft robot (**a**) caused by increasing the air pressure inside the air chamber and tendon tension (**b**) Cross-section of the soft robot, illustrating the chamber undergoing pressurization and the tendon being pulled.

During the FE simulations, hyperelastic silicone material, based on Table 1, was assigned to the soft robot by considering the large deformation of the material. The soft robot was considered to be a cantilever beam using Dirichlet and Neumann boundary conditions to establish the boundary conditions, where the displacements and rotations at the base of the soft robot were fixed. To simulate the robot’s deformation, internal air pressure was applied to the inner surface of one air chamber as well as the cap of the air chamber. Simultaneously, a tendon force was exerted on the tip of the soft robot in the opposite direction. This loading configuration made the soft robot bend in a 2D plane. In this study, all simulations were performed using an Intel Core (TM) i7-10700K central processing unit (CPU) at 3.80 GHz with 16.0 GB random-access memory (RAM). The deformation of the soft robot is illustrated in Figure 6a until it reaches 90°. Once it reaches 90°, the cross-section deformation is shown in Figure 6b.

## 3. Results and Discussion

The RSO technique was employed to generate a total of 45 design experiments, which were based on the geometrical constraints and variations of the applied loadings. Throughout the simulations, the air pressure was incrementally increased from 12 kPa to 16 kPa while simultaneously applying a tendon force ranging from 40 mN to 60 mN to the tip of the soft robot. A comprehensive overview of all 45 design experiment points, along with their respective geometries, pressures, tendon forces, and corresponding values for the goal function, can be found in Table 2. Subsequently, in adherence with the clinical requirements discussed in Section 2.1, design points with bending angles of less than 90° were excluded from further consideration. This step ensured that only feasible design point options were pursued. The next objective was to minimize the goal function presented in (1) by utilizing the Genetic Algorithm (GA) method. The aim was to identify the optimum design point for the soft robot’s tip to achieve a bending angle of 90°. The optimized values derived from this process were subsequently utilized to prototype the hybrid-driven soft robot. These optimized values can be found in summarized form in Table 3.

**Figure 7:**
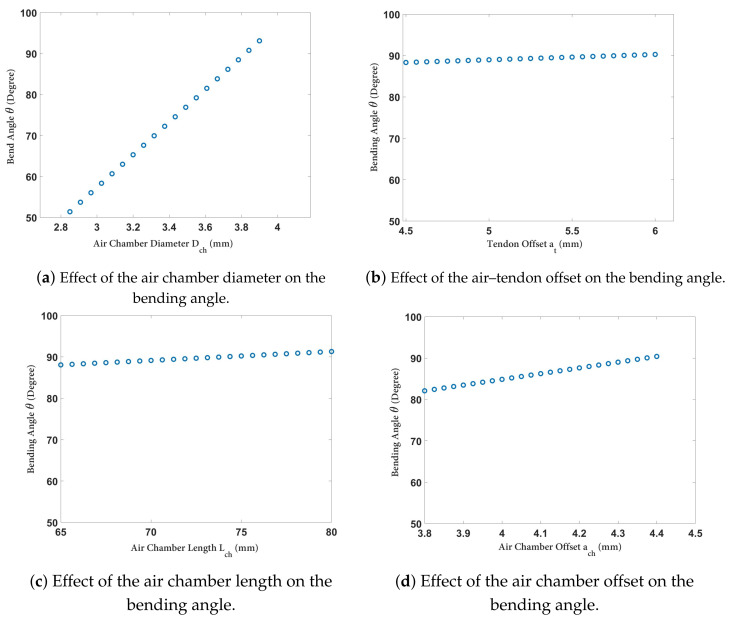
Variation in the bending angle of the soft robot vs. (**a**) the air chamber diameter, $D_{Ch}$ , (**b**) tendon offset $a_{t}$ , (**c**) air chamber length $L_{Ch}$ , and- (**d**) air chamber offset $a_{Ch}$ .

Figure 7a illustrates the variation in the bending angle ( θ ) with respect to the diameter of the air chamber. As observed, in accordance with our expectations, an increase in the diameter of the air chamber led to a proportional increase in the area term (*A*) in the equation for the PA . Consequently, this resulted in a larger moment around the central axis of the soft robot, causing it to bend further. Similarly, Figure 7b demonstrates that increasing the offset of the tendon passages from the center also increased the bending angle. Due to the minimum requirement of a 1 mm wall thickness, the maximum value of $a_{t}$ was limited to 6 mm. Next, Figure 7c illustrates the variation in the bending angle relative to the length of the air chamber ( $L_{Ch}$ ). Additionally, Figure 7d indicates that the effect of the air chamber offset on the bending angle was minimal. It should be noted that increasing $a_{Ch}$ negatively impacted the “ballooning effect” of the soft robot. It is crucial to consider the placement of the air chamber, which should not be close to the edge of the cross-section to avoid unnecessary radial expansion.

**Figure 8:**
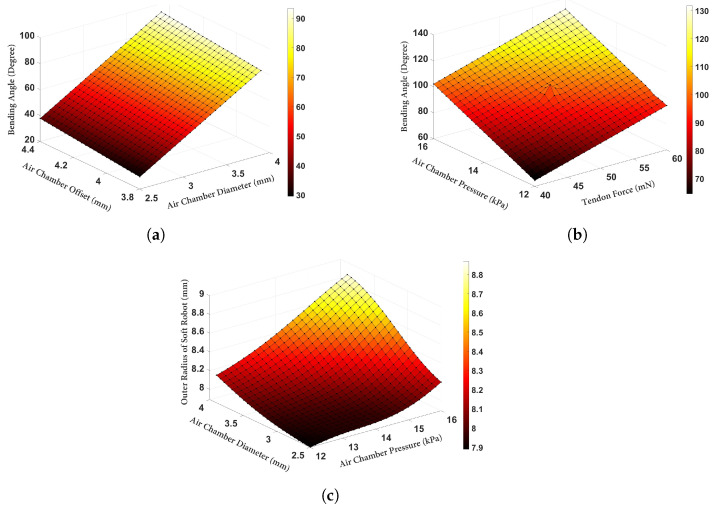
Deformation of the soft robot with the optimized parameters (**a**) variation in the bending angle vs. the air chamber diameter and offset. (**b**) Variation in the bending angle vs. the tendon force and air chamber pressure. (**c**) Variation in the outer radius of the soft robot vs. the air chamber pressure and diameter.

Figure 8a displays the variation in the bending angle ( θ ) as the air chamber diameter and offset were changed. As can be observed, the air chamber diameter played a significant role in increasing the bending angle. Although increasing the air chamber diameter reduced the required pressure for a certain amount of bending, it also resulted in an increased radial expansion. This antagonistic feature in (1) between the first and second terms could be utilized to identify the optimal geometry for minimizing the goal function. Furthermore, Figure 8b demonstrates the variation in the bending angle when the loading inputs (i.e., tendon force and air chamber pressure) were changed, while the other input parameters were set to the optimized values based on Table 3. Finally, Figure 8c illustrates the outer radial expansion of the soft robot when the air chamber pressure and diameter were varied. It is evident that, even with the use of the optimized value, the radius of the cross-section increased by approximately 25%.

**Figure 9:**
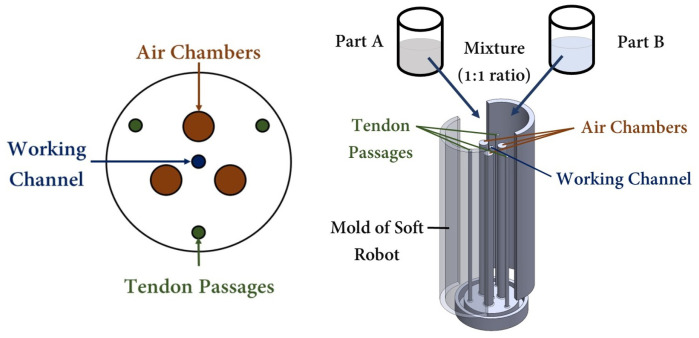
Mold design of the hybrid air–tendon-driven soft robot with a central working channel.

**Figure 10:**
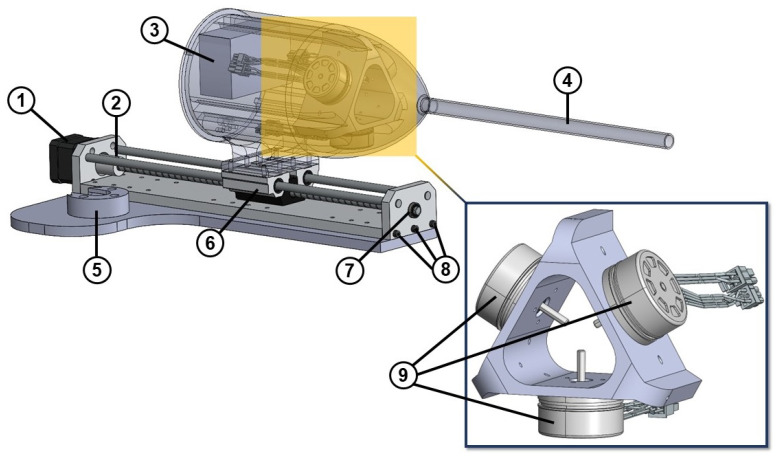
Linear actuator of the soft robot: ① NEMA 17 stepper motor, ② shaft coupler, ③ EPOS4 3-axes digital positioning controller of the motors, ④ silicone tube, ⑤ holder of the robotic arm, ⑥ double bearing and lead screw, ⑦ bearing, ⑧ screws, and ⑨ brushless DC motor with Hall sensors.

To demonstrate the performance of a soft robot with optimized values in accordance with the proposed study for robot-assisted intervention purposes, a prototype of a hybrid-driven soft robot was created. This involved the utilization of a cylindrical mold with internal air chambers, tendon passes, and a central working channel, which was rapidly fabricated using a 3D printer (Replicator+, MakerBot, New York, NY, USA). Figure 9 provides an illustration of the mold design employed for prototyping the hybrid-driven soft robot. The mold was designed in three separate parts to facilitate the easy removal of the soft robot body. Upon successful completion of the mold printing process, two parts of Ecoflex 00-50 silicone were mixed in a 1:1 ratio and subsequently placed in a vacuum chamber for degassing. After eliminating any excess bubbles from the silicon mixture, it was poured into the mold and allowed to cure at room temperature for a period of 12 h. Once the silicone had fully dried, the soft robot was detached from the mold. Subsequently, a silicone tube was affixed to the base of the soft robot, as depicted in Figure 10 ④, to attach it to the tendon module. The material of the tube is stiffer than the distal tip of the soft robot, so by increasing the air pressure inside the chambers, only the soft robot at the tip will be inflated.

In addition, the linear actuator utilized for the insertion and retraction of the soft robot is illustrated in Figure 10. This was achieved through the installation of a stepper motor (Nema 17) that was affixed to the linear actuator’s rail. Moreover, the setup incorporated three brushless DC motors (Maxon, EC 45 flat ϕ 42.8 mm, 60 W with Hall sensors, Sachseln, Switzerland) along with a digital positioning controller (Maxon, EPOS4 Compact 24/5 Ether CAT 3-axes, Sachseln, Switzerland) to provide the necessary tendon force at the soft robot’s tip. To enable the soft robot’s rotation, a specially designed holder was employed to attach the linear actuator to the CRS robotic arm.

**Figure 11:**
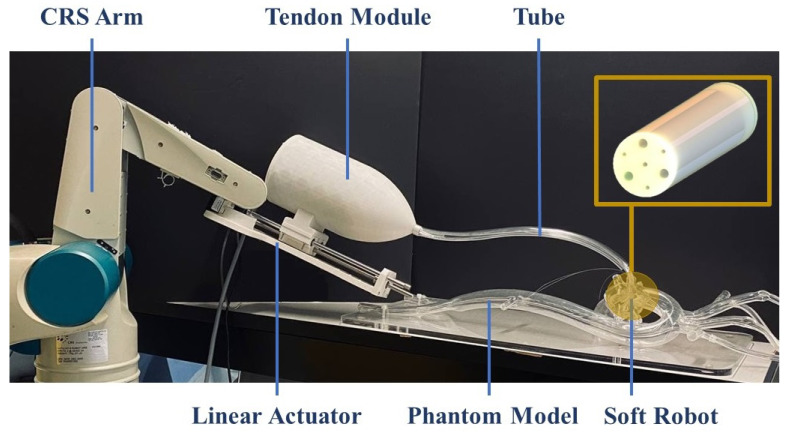
Integrated hybrid-driven soft robot into the CRS robotic arm and the phantom model.

Figure 11 illustrates the integration of the optimized soft robot prototype with the CRS robotic arm. The figure depicts the soft robot as an end effector at the tip of a surgical instrument (i.e., a catheter), the tube containing tendons and air pathways, and the placement of motors inside the tendon module. The CRS robotic arm facilitates the positioning of the soft robot in the phantom model, while the rotation of the tip is achieved through the manipulation of tendons. Optimizing the geometry of the soft robot minimizes radial expansion during inflation, thereby reducing unnecessary expansions and enabling smoother navigation of surgical tools through tortuous paths, which are represented here using the phantom model. The synergy of these components in a hybrid air–tendon system results in a versatile and effective system and facilitates control during robot-assisted interventions.

## 4. Conclusions

This paper presented a comprehensive study on the design optimization of a hybrid-driven soft robot for use in RAMIS. The proposed FEA-based method enables the optimization of the soft robot’s geometry, considering various design constraints specific to different clinical tasks. The optimized geometry aims to reduce the actuation effort, improve the control, and minimize unnecessary radial expansion. To this end, the study employed a cylindrical soft robot with three air chambers, three tendons, and a central working channel. The dimensions of the soft robot were iteratively optimized using the RSO module in ANSYS software. The material properties of the silicon used in the soft robot were determined through experimental testing and hyperelastic modeling. The optimized geometry led to an enhanced performance and reduced cross-sectional expansion of the soft robot. These improvements resulted in decreased actuation efforts and improved the robot’s ability to perform delicate and precise interventions. Additionally, the study validated the optimization methodology by fabricating a prototype of the optimized soft robot and deploying it in a phantom model that closely mimicked the anatomical environment encountered during minimally invasive interventions. The practical evidence obtained from this realistic setting further confirmed the effectiveness of the optimization approach. The incorporation of FEA simulations, design constraints, and a multi-objective goal function allowed for the systematic refinement of the soft robot’s design. Future work will focus on additional optimizations, considering factors such as multi-materials and constraints, to enhance the soft robot’s versatility and expand its applications in RAMIS. Specifically, the study suggests that position control experiments should be conducted to achieve the desired trajectories for the soft robot’s tip and the results should be compared before and after optimization. Additionally, the ability to adjust the stiffness of the soft robot during manipulation is of high clinical importance and could be achieved using the hybrid-driven mode suggested in the current study. Moreover, synthetic data generated by the simulations could later be used to train a neural network to model a hybrid actuated soft robot, similar to the work done by S. Terrile et al. [65]. Finally, to overcome the manufacturing challenges mentioned in the study, advanced fabrication technologies, such as multi-material 3D printing, are promising.

## Figures and Tables

**Table 1:** Material constants of the Yeoh model for Ecoflex 00-50.

Model Parameters	$C_{10}$ (MPa)	$C_{20}$ (MPa)	$C_{30}$ (MPa)
With Compression	0.01516	0.00010	$-7.39\times 10^{-8}$
Without Compression [61]	0.01385	0.00011	$-8.76\times 10^{-8}$

**Table 2:** Design points of the experiments.

Number	$D_{Ch}$	$a_{Ch}$	$a_{t}$	$L_{Ch}$	$F_{t}$	$P_{Ch}$	θ	Goal Function		
	(mm)	(mm)	(mm)	(mm)	(mN)	(kPa)	(Degree)	w=0.05	w=0.5	w=0.95
1	3.605	4.273	4.816	68.165	55.780	15.156	114.742	0.274	0.331	0.387
2	3.900	4.100	5.250	72.500	50.000	14.000	108.421	0.211	0.267	0.323
3	3.605	3.927	5.684	68.165	55.780	15.156	107.886	0.183	0.283	0.382
4	3.605	4.273	4.816	76.835	44.220	15.156	105.432	0.223	0.279	0.336
5	3.605	4.273	5.684	76.835	55.780	15.156	104.453	0.151	0.266	0.381
6	3.605	3.927	4.816	76.835	55.780	15.156	99.472	0.102	0.240	0.378
7	3.605	3.927	5.684	76.835	44.220	15.156	98.431	0.157	0.245	0.332
8	3.200	4.100	5.250	72.500	50.000	16.000	89.935	0.103	0.242	0.380
9	3.605	4.273	5.684	68.165	44.220	15.156	85.283	0.132	0.231	0.331
10	3.200	4.100	5.250	72.500	60.000	14.000	81.807	0.045	0.203	0.360
11	3.605	4.273	4.816	76.835	55.780	12.844	81.316	0.095	0.203	0.310
12	3.605	3.927	5.684	76.835	55.780	12.844	80.006	0.054	0.181	0.307
13	3.605	3.927	4.816	68.165	44.220	15.156	79.828	0.092	0.211	0.329
14	3.605	4.273	5.684	68.165	55.780	12.844	74.464	0.047	0.177	0.307
15	3.200	4.400	5.250	72.500	50.000	14.000	73.255	0.064	0.189	0.315
16	3.605	3.927	4.816	68.165	55.780	12.844	71.309	0.036	0.171	0.307
17	2.795	4.273	4.816	76.835	55.780	15.156	70.477	0.073	0.225	0.377
18	2.795	4.273	5.684	68.165	55.780	15.156	69.322	0.043	0.209	0.375
19	3.200	4.100	6.000	72.500	50.000	14.000	67.575	0.047	0.180	0.314
20	3.200	4.100	5.250	72.500	50.000	14.000	66.894	0.044	0.179	0.314
21	3.200	4.100	5.250	80.000	50.000	14.000	66.670	0.054	0.184	0.314
22	2.795	3.927	5.684	76.835	55.780	15.156	66.501	0.029	0.202	0.374
23	3.200	4.100	4.500	72.500	50.000	14.000	65.859	0.044	0.179	0.314
24	3.200	4.100	5.250	65.000	50.000	14.000	64.635	0.043	0.178	0.314
25	2.795	3.927	4.816	68.165	55.780	15.156	61.770	0.027	0.201	0.374
26	3.605	4.273	4.816	68.165	44.220	12.844	60.188	0.060	0.160	0.259
27	2.795	4.273	5.684	76.835	55.780	12.844	60.007	0.030	0.168	0.306
28	3.200	3.800	5.250	72.500	50.000	14.000	59.712	0.033	0.173	0.313
29	3.605	3.927	5.684	68.165	44.220	12.844	59.630	0.047	0.152	0.258
30	3.605	4.273	5.684	76.835	44.220	12.844	58.944	0.046	0.152	0.258
31	2.795	4.273	4.816	68.165	55.780	12.844	57.122	0.029	0.168	0.306
32	2.795	3.927	5.684	68.165	55.780	12.844	55.601	0.021	0.163	0.306
33	3.605	3.927	4.816	76.835	44.220	12.844	54.974	0.035	0.146	0.258
34	2.795	3.927	4.816	76.835	55.780	12.844	53.474	0.020	0.163	0.306
35	2.795	4.273	5.684	76.835	44.220	15.156	53.446	0.045	0.186	0.326
36	3.200	4.100	5.250	72.500	50.000	12.000	53.213	0.028	0.143	0.258
37	3.200	4.100	5.250	72.500	40.000	14.000	51.423	0.042	0.159	0.276
38	2.795	4.273	4.816	68.165	44.220	15.156	50.070	0.048	0.187	0.327
39	2.795	3.927	5.684	68.165	44.220	15.156	46.476	0.029	0.178	0.326
40	2.795	3.927	4.816	76.835	44.220	15.156	45.246	0.027	0.176	0.326
41	2.500	4.100	5.250	72.500	50.000	14.000	44.326	0.021	0.167	0.313
42	2.795	4.273	5.684	68.165	44.220	12.844	39.325	0.026	0.142	0.257
43	2.795	4.273	4.816	76.835	44.220	12.844	38.646	0.035	0.146	0.258
44	2.795	3.927	5.684	76.835	44.220	12.844	37.541	0.020	0.139	0.257
45	2.795	3.927	4.816	68.165	44.220	12.844	33.435	0.019	0.138	0.257

**Table 3:** Optimized dimensions for the hybrid-driven soft robot.

Parameters	Optimized Values (mm)		
	w=0.05	w=0.5	w=0.95
$D_{ch}$	3.49	3.82	3.89
$L_{ch}$	72.96	74.45	77.33
$a_{ch}$	3.80	4.37	4.36
$a_{t}$	4.57	5.85	5.72

## Data Availability

Data are contained within the article.

## Abbreviations

The following abbreviations are used in this manuscript:

RAMIS	Robot-Assisted Minimally Invasive Surgery
FEA	Finite Element Analysis
MIS	Minimally Invasive Surgery
RSO	Response Surface Optimization
EAP	Electroactive Polymer
FOV	Field of View
DOF	Degrees of Freedom
UTM	Universal Testing Machine
2D	Two-Dimensional
PCC	Piecewise Constant Curvature
ISO	International Organization for Standardization
CPU	Central Processing Unit
RAM	Random-Access Memory
GA	Genetic Algorithm
